# Thymosin α1 alleviates pulpitis by inhibiting ferroptosis of dental pulp cells

**DOI:** 10.1038/s41368-025-00394-4

**Published:** 2025-10-14

**Authors:** Jie Wu, Qimei Gong, Wenxuan Liu, Aijia Chen, Zekai Liao, Yihua Huang, Wenkai Jiang, Zhongchun Tong

**Affiliations:** 1 Hospital of Stomatology, Sun Yat-sen University, Guangzhou, China; 2 Guangdong Provincial Key Laboratory of Stomatology, Sun Yat-sen University, Guangzhou, China; 3 Guanghua School of Stomatology, Sun Yat-sen University, Guangzhou, China; 4 State Key Laboratory of Oral & Maxillofacial Reconstruction and Regeneration & National Clinical Research Center for Oral Diseases & Shaanxi Key Laboratory of Stomatology, Department of Operative Dentistry & Endodontics, School of Stomatology, Fourth Military Medical University, Xi’an, China

**Keywords:** Pulpitis, Experimental models of disease

## Abstract

Tooth pulpitis is a prevalent oral disorder. Understanding the underlying mechanisms of pulpitis and developing effective treatment strategies hold great significance. Ferroptosis has recently emerged as a new form of cell death, but the role of ferroptosis in pulpitis remains largely unknown. In our study, single-cell RNA sequencing (scRNA-seq) was used to identify cellular heterogeneity between 3 pulpitis tissue and 3 healthy pulp tissue, and explored ferroptosis occurrence in pulpitis tissue and inflamed dental pulp cells (DPCs). In scRNA-seq, 40 231 cells (Pulpitis: 17 814; Healthy pulp: 22 417) were captured, and visualized into 12 distinct cell clusters. Differentially expressed ferroptosis-related genes (DE-FRGs) were almost presented in each cluster in pulpitis vs healthy pulp. ROS and Fe^2+^ levels significantly rose, and immunohistochemistry showed low expression of GPX4 and high expression of PTGS2 in pulpitis. In LPS-stimulated DPCs, thymosin α1 increased the expression of GPX4 and FTL, and decreased expression of TNF-α, IL-1β, IL-6, and Fe^2+^ levels. In rat pulpitis models, both prothymosin α (PTMA, precursor of thymosin α1) gelatin sponge placed at the hole of pulp (LPS-P(gs)) and PTMA injection in pulp (LPS-P(i)) significantly reduced infiltration of inflammatory cells and expression of PTGS2, and increased the expression of GPX4. In RNA sequencing, the expression of DE-FRGs were reversed when thymosin α1 were added in LPS-stimulated DPCs. Collectively, single-cell atlas reveals cellular heterogeneity between pulpitis and healthy pulp, and ferroptosis occurrence in pulpitis. Thymosin α1 may reduce ferroptosis in DPCs to alleviate pulpitis and thus potentially has the ability to treat pulpitis.

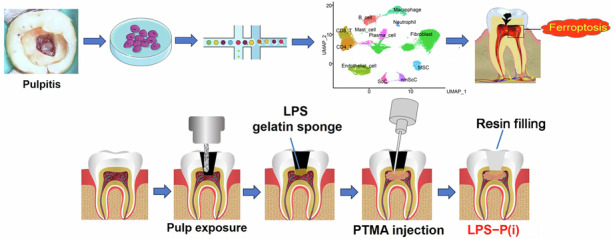

## Introduction

Tooth pulpitis is a common oral disorder, which can lead to intense pain and significant discomfort. Understanding the underlying mechanisms of pulpitis and developing effective treatment strategies are of great significance. The etiology of pulpitis is mainly attributed to dental caries, dental trauma, and bacterial invasion.^1–3^ When the enamel and dentin are damaged, bacteria can enter the dental pulp, leading to an inflammatory response. The pathological features of pulpitis include congestion, edema, and inflammatory cell infiltration of the dental pulp. As the inflammation progresses, it can cause necrosis of the dental pulp tissue.^4^ There are remarkable changes in dental pulp cells in pulpitis. Inflammatory cells such as macrophages, neutrophils, T cells and B cells infiltrate the dental pulp, release inflammatory mediators and cause damage to the pulp cells. At the same time, the activity and function of pulp cells themselves also change, affecting the normal physiological processes of dental pulp.^2,5–7^

Recently, ferroptosis has emerged as a new form of cell death that is clearly distinguishable from apoptosis, pyroptosis, and necrosis. It is marked by the accumulation of iron-dependent lipid peroxides. These peroxides cause damage to the cell membrane and ultimately result in cell death.^8,9^ Ferroptosis can contribute to inflammation.^10–12^ Recent studies show that the activation of inflammation, particularly the activation of multiple inflammation-related signaling pathways, can induce ferroptosis.^12–14^ When cells undergo ferroptosis, the release of damage-associated molecular patterns (DAMPs) can trigger an immune response. DAMPs can activate immune cells like macrophages and dendritic cells, thereby resulting in the production of pro-inflammatory cytokines and chemokines. This can amplify the inflammatory response and contribute to the progression of various diseases. Glutathione peroxidase 4 (GPX4) is the key regulator of ferroptosis, and prostaglandin-endoperoxide synthase 2 (PTGS2) is a marker of ferroptosis occurrence.^15,16^ Currently, there is relatively little research on ferroptosis in dental pulp inflammation. Xie et al. identified 72 differentially expressed ferroptosis-related genes (DE-FRGs) by comparing microarray and sequencing data for seven pulpitis samples and five healthy dental pulp samples.^17^ However, despite the identification of these DE-FRGs, the occurrence of ferroptosis in pulpitis still needs to be further validated.

Thymosin α1, a polypeptide composed of 28 amino acids, is a cleavage product of prothymosin α (PTMA), which consists of 109 amino acid residues and is cleaved by asparaginyl endopeptidase.^18^ Thymosin α1 has the ability to enhance the responses of T cells, natural killer cells, dendritic cells, and antibodies.^19–21^ It can also modulate the production of cytokines and chemokines and block steroid-induced apoptosis of thymocytes.^22^ Owing to its biological diversity, thymosin α1 has attracted increasing attention in recent years and has been utilized in the treatment of cancers and infectious diseases.^20,23–25^ In a previous study, thymosin α1 was discovered to potentially lower the expressions of inflammatory cytokines and macrophage osteoclastogenesis, thereby providing a foundation for targeted therapeutic strategies for apical periodontitis.^26^

Dental pulpitis is a common inflammatory disease of teeth. Thymosin α1, a well-known polypeptide with immunoregulatory effects, has not been reported on in the context of treating dental pulpitis. In this study, single-cell RNA sequencing was first employed to analyze the cell types in inflammatory dental pulp and explore the occurrence of ferroptosis in dental pulp cells. Secondly, in vitro and in vivo experiments as well as RNA sequencing were conducted to investigate whether thymosin α1 inhibits ferroptosis in inflammatory dental pulp cells and pulp tissue to restore pulpitis.

## Results

### ScRNA-seq unveils the cellular diversity and heterogeneity of healthy pulp and inflammtory pulp

**Fig. 1:**
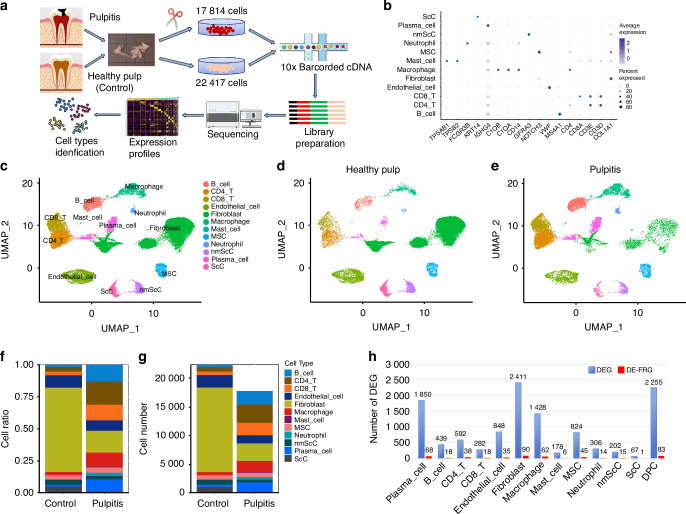
Single-cell RNA sequencing of pulpitis and healthy pulp. **a** Diagram of single-cell RNA sequencing (ScRNA-seq) of pulpitis and healthy pulp (Control). **b** Marker genes of different cell types. **c** Uniform manifold approximation and projection (UMAP) plots of 40 231 cells revealed 12 main cell types in 3 pulpitis samples and 3 healthy pulp samples. UMAP of healthy pulp (**d**) and pulpitis (**e**). **f** Proportions of the different cell types in control pulp and pulpitis. **g** The actual number of each type of cells in control pulp and pulpitis. **h** The blue column represents the number of differentially expressed genes (DEGs) between pulpitis and healthy pulp; the red column represents the number of differentially expressed ferroptosis-related genes (DE-FRGs)

To analyze cellular heterogeneity and explore ferroptosis in each type of cell in inflammatory dental pulp, three healthy dental pulps and three inflammatory dental pulps were harvested. The six pulp tissue samples were dissociated to obtain single-cell suspensions and then underwent single-cell RNA sequencing (Fig. 1a). After quality control, the transcriptomes of 40 231 cells (17 814 from pulpitis samples and 22 417 from healthy pulp samples) were obtained. These cells were subsequently visualized into 12 distinct cell clusters, including macrophage, neutrophil, mast cell, B cell, CD^4+^ T cell, CD^8+^ T cell, plasma cell, fibroblast, endothelial cell, mesenchymal stem cell (MSC), Schwann cell (ScC), and nonmyelinating ScC (nmScC), by generating uniform manifold approximation and projection (UMAP) plots based on hierarchical clustering and established lineage-specific marker genes (Fig. 1b, c). The 12 cell clusters in normal pulp and inflammatory pulp were shown in Fig. 1d, e. Fibroblasts dominated the healthy pulp but significantly decreased in pulpitis. Immune cells such as macrophage, neutrophil, mast cell, B cell, CD^4+^ T cell, CD^8+^ T cell, and plasma cell markedly increased in pulpitis (Fig. 1f, g). In differentially expressed genes (DEGs) between pulpitis and healthy pulp, the number of DEGs in fibroblasts was also the highest among all cell types. The DEGs in each cell cluster were intersected with 484 genes related to ferroptosis according to the FerrDB database (Fig. 1h). The abundance of ferroptosis-related genes present in the DEGs between pulpitis and healthy pulp indicates the occurrence of ferroptosis in pulpitis.

### Ferroptosis in pulpitis

**Fig. 2:**
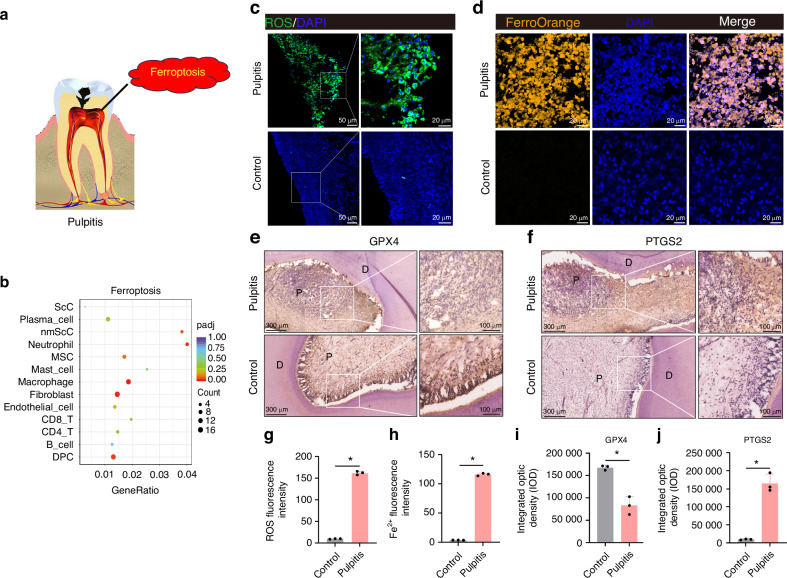
Evaluation of ferroptosis in pulpitis. **a** Pattern of ferroptosis in pulpitis. **b** KEGG enrichment analysis of ferroptosis in single-cell RNA sequencing between pulpitis and healthy pulp tissue. **c** The levels of ROS in pulpitis and control pulp tissue were measured by the DCFH-DA fluorescent probe (green). **d** Intracellular Fe^2+^ levels in pulpitis and control pulp tissue were detected with the FerroOrange fluorescent probe (orange). Scale bars: 20 μm. Immunohistochemistry of GPX4 (**e**) and PTGS2 (**f**) in human pulpitis and control pulp tissue. D: dentin; P: tooth pulp. **g** Quantitative analysis of ROS fluorescence intensity in pulpitis and control pulp tissue. **h** Quantitative analysis of Fe^2+^ fluorescence intensity in pulpitis and control pulp tissue. Cell nuclei were stained with DAPI (blue). Integrated optical density (IOD) of GPX4 (**i**) and PTGS2 (**j**) in human pulpitis and control pulp tissue by Image J Pro software. Values are represented as mean ± SD. *P < 0.05

To further investigate ferroptosis in pulpitis (Fig. 2a), we summarized ferroptosis pathway in 12 cell types and DPCs in scRNA-seq data of pulpitis vs healthy pulp (Control). Ferroptosis pathway were significantly enriched in fibroblast, macrophage, MSC, neutrophil cell, nmScC and DPC (Fig. 2b). We further investigated some pathway related with ferroptosis, such as oxidative phosphorylation, p53, MAPK, PI3K-Akt, mTOR, and AMPK signaling pathway (Figure. S1a-f).^27–30^ Oxidative phosphorylation was the most significantly enriched signaling pathway, indicating its substantial contribution to ferroptosis in pulpitis. ROS and Fe^2+^ were two key indicators of ferroptosis. To evaluate the occurrence of ferroptosis in pulpitis, ROS and Fe^2+^ levels in pulp tissue were measured by the DCFH-DA fluorescent probe (Green) and FerroOrange fluorescent probe (Orange). We found that ROS and Fe^2+^ levels were evidently higher in pulpitis than in healthy pulp tissue (Fig. 2c, d, g, h). Furthermore, in immunohistochemistry of GPX4 and PTGS2, more inflammatory cells were present in pulpitis. GPX4 showed significantly lower expression in pulpitis than in control pulp (Fig. 2e, i), and conversely, PTGS2 was higher expressed in pulpitis than in control pulp (Fig. 2f, j).

### The effect of thymosin α1 on the ferroptosis of dental pulp cells

**Fig. 3:**
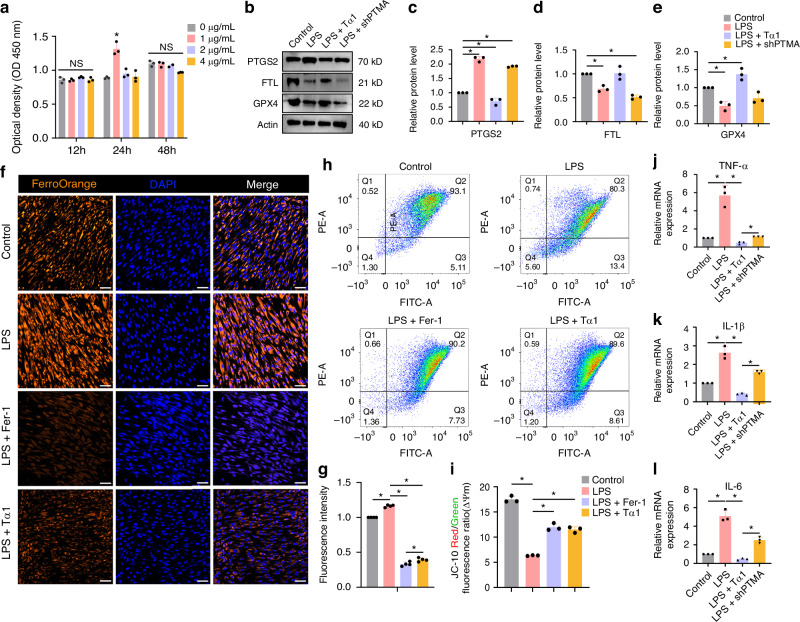
The effect of thymosin α1 on the ferroptosis of dental pulp cells (DPCs). **a** CCK8 assay: The effect of different concentrations of Tα1 on the growth of DPCs. **b** The expression of PTGS2, FTL, and GPX4 proteins was evaluated using western blot in four groups of DPCs including Control group, LPS-stimulated DPCs (LPS group), LPS-stimulated DPCs with Tα1 addition (LPS + Tα1 group), and LPS-stimulated DPCs with *Ptma* gene silenced (LPS + shPTMA group). The statistical analysis of PTGS2 (**c**), FTL (**d**), and GPX4 (**e**) proteins expression in four groups of DPCs. (**f**) Intracellular Fe^2+^ levels in DPCs were measured by the FerroOrange fluorescent probe (orange). Cell nuclei were stained with DAPI (blue). A ferroptosis inhibitor, ferrostatin-1, was added into LPS-stimulated DPCs (LPS + Fer-1). Scale bars: 50 μm. **g** Quantitative analysis of the FerroOrange fluorescent intensity of the four groups of DPCs. Bars represent means ± SD. **h** Flow cytometry of JC-10 of the four groups. Accumulation of JC-10 aggregates in the mitochondrial matrix can be detected by red fluorescence (Ex/Em: 540/590 nm). At mitochondrial depolarization, JC-10 diffuses out of the mitochondria and returns to its monomeric form which exhibits green fluorescence (Ex/Em: 490/525 nm). **i** Statistical analysis of JC-10 red/green fluorescence ratio among the four groups. The expression of the inflammatory factor TNF-α (**j**), IL-1β (**k**), and IL-6 (**l**) among the four groups of DPCs. *P < 0.05. NS: no statistical difference

In light of ferroptosis in pulpitis, we further investigated the occurrence of ferroptosis in dental pulp cells stimulated with lipopolysaccharide (LPS). GSEA of ferroptosis was shown in RNA sequencing of DPCs stimulated with LPS, and 68 genes were listed (Figure. S2a, b). GPX4 showed low expression in LPS-stimulated DPCs as indicated by a heatmap. Next, we aimed to evaluate whether thymosin α1 alleviated ferroptosis of DPCs. In a CCK8 assay, 1 μg/mL Tα1 promoted DPCs proliferation at 24 h and was used to test in the following experiments (Fig. 3a). LPS stimulation decreased the expression of GPX4 and FTL protein, and increased the expression of PTGS2 protein in DPCs. The addition of Tα1 increased the expression of GPX4 and FTL, and decreased the expression of PTGS2. After the *Ptma* gene was silenced in DPCs, the expression of the three proteins were reversed in LPS-stimulated DPCs (Fig. 3b). The statistical differences were shown in the expression of PTGS2 (Fig. 3c), FTL (Fig. 3d) and GPX4 (Fig. 3e) proteins among the four groups of DPCs (Control, LPS, LPS + Tα1 and LPS+shPTMA). In an intracellular Fe^2+^ assay, FerroOrange fluorescence in DPCs significantly increased under LPS stimulation. Ferrostatin-1, a ferroptosis inhibitor, significantly inhibited the FerroOrange fluorescent expression of LPS-stimulated DPCs. Similarly, Tα1 also decreased intracellular Fe^2+^ level in LPS-stimulated DPCs (*P* < 0.05, Fig. 3f, g). Changes in mitochondrial membrane potential are a key indicator of ferroptosis. JC-10 can be used to check fluorescence intensity changes to determine the degree of mitochondrial membrane potential alteration. The JC-10 red/green fluorescence ratio significantly decreased in LPS-stimulated DPCs in comparison with the control DPCs, and Tα1, like Ferr-1, significantly increased JC-10 red/green fluorescence ratio in LPS-stimulated DPCs (*P* < 0.05, Fig. 3h, i). These results indicated that Tα1 reduced ferroptosis of LPS-stimulated DPCs. Furthermore, Tα1 lowered the expression of TNF-α, IL-1β, and IL-6 in LPS-stimulated DPCs, and the expression of the three factors increased in LPS-stimulated shPTMA DPCs (*P* < 0.05, Fig. 3j–l).

### The effect of PTMA on the pulpitis of rats

**Fig. 4:**
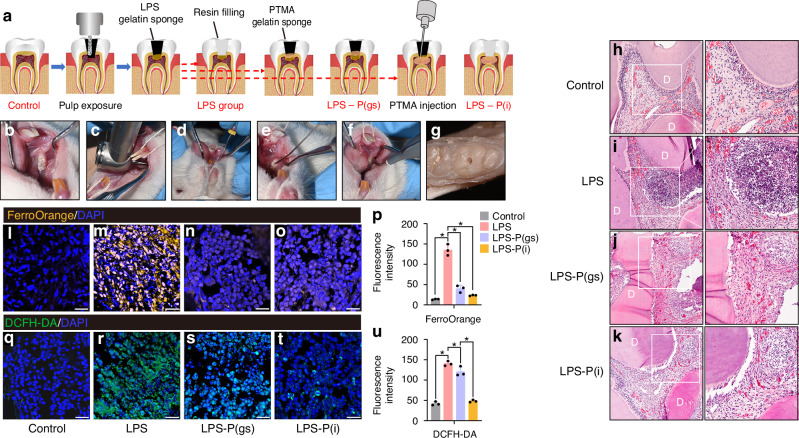
The effect of PTMA on the pulpitis of rat. **a** Diagrammatic sketch of PTMA sealed in the teeth of rats with pulpitis; **b**–**g** The operation procedure for inducing pulpitis in rats. Hematoxylin and eosin (H&E) staining images of demineralized and paraffin-embedded dental pulp tissue sections in the four groups. (**h**) Control pulp; (i) LPS group: LPS gelatin sponge placed on the exposed pulp cavity; (**j**) LPS-P(gs) group: PTMA gelatin sponge placed above the LPS gelatin sponge; (**k**) LPS-P(i) group: LPS gelatin sponge were placed on the exposed pulp, and PTMA were then injected into pulp cavity. Scale bars: 100 μm. D: dentin. Right images are partial magnifications of left images in each group. Intracellular Fe^2+^ levels in Control group (**l**), LPS group (**m**), LPS-P(gs) group (**n**), and LPS-P(i) group (**o**) of pulp tissue were detected with the FerroOrange fluorescent probe (orange). The levels of ROS in Control group (**q**), LPS group (**r**), LPS-P(gs) group (**s**), and LPS-P(i) group (**t**) were measured by the DCFH-DA fluorescent probe (green). Cell nuclei were stained with DAPI (blue). Scale bars: 20 μm. Quantitative analysis of the FerroOrange (**p**) and DCFH-DA (**u**) fluorescent intensity of the four groups of teeth pulp. Bars represent means ± SD. *P < 0.05. PTMA: Prothymosin-α; LPS: Lipopolysaccharide

**Fig. 5:**
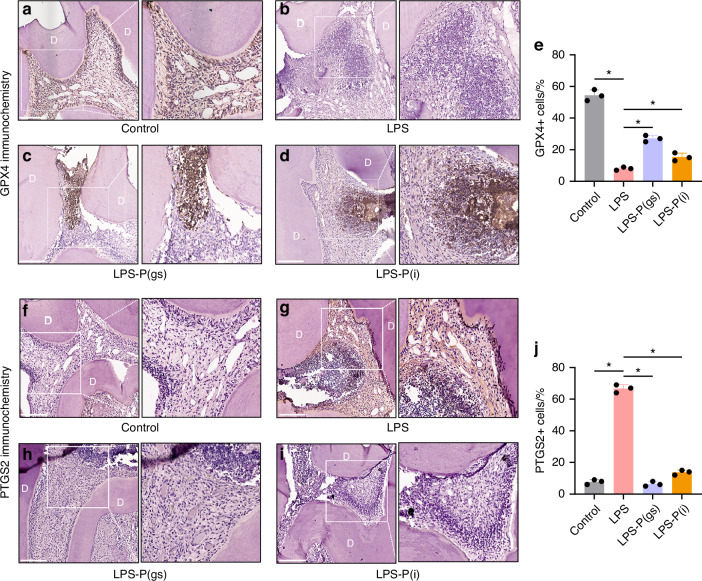
Immunohistochemical staining of GPX4 (**a**–**e**) and PTGS2 (**f**–**j**) in pulp tissue of rats in the Control, LPS, LPS-P(gs), and LPS-P(i) groups. Percentages of GPX4 positive cells (**e**) and PTGS2 positive cells (**j**) in each group were shown (Means ± SD, 3 rats per group). Scale bars: 100 μm, *P < 0.05. D: Dentin; PTMA: Prothymosin-α; LPS: Lipopolysaccharide. Right of each image are partial magnifications of the left images in each group

A rats’ pulpitis model was established to evaluate the effect of prothymosin α (PTMA) treatment (Fig. 4a–g). The infiltration of numerous inflammatory cells indicated the successful construction of the LPS-induced pulpitis model in rats (Fig. 4i) in comparison with the control pulp (Fig. 4h). Both placing PTMA gelatin sponge at the pulp hole (LPS-P(gs)) and injecting PTMA into the pulp (LPS-P(i)) significantly reduced the infiltration of inflammatory cells (Fig. 4j, k). Fe^2+^ levels of rat pulp were significantly higher in LPS group than in control group, but Fe^2+^ levels showed significantly reduction in LPS-P(gs) and LPS-P(i) groups (*P* < 0.05, Fig. 4l–p). Similarly, ROS levels of the four groups of rat pulp showed identical results (*P* < 0.05, Fig. 4q–u). GPX4 is a key regulator in ferroptosis by reducing lipid peroxidation and protecting cells from iron-dependent oxidative damage. In immunohistochemical staining, the expression of GPX4 was significantly lower in LPS-induced pulpitis than in the control, indicating the occurrence of ferroptosis in pulpitis (*P* < 0.05, Fig. 5a, b, e). GPX4 significantly increased when PTMA was applied in LPS-induced pulpitis, including in the LPS-P(gs) group and the LPS-P(i) group (*P* < 0.05, Fig. 5c–e). PTGS2 is often considered a reliable marker for ferroptosis. The presence of PTGS2 indicates the occurrence and progression of ferroptosis. LPS-induced pulpitis showed higher expression of PTGS2 in comparison with control, and the use of PTMA significantly decreased PTGS2 expression (*P* < 0.05, Fig. 5f–j).

### RNA-seq of inflammatory dental pulp cells with thymosin α1 treatment

**Fig. 6:**
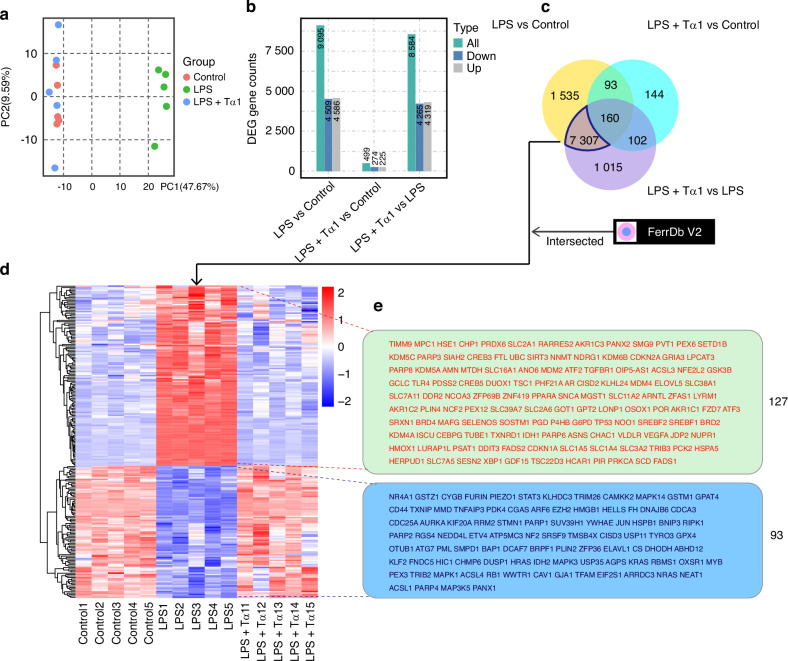
RNA-seq of dental pulp cells (DPCs) with LPS stimulation and thymosin α1 treatment. RNA-seq of DPCs were divided into three groups: Control, LPS-stimulated DPCs (LPS), and LPS-stimulated DPCs with thymosin α1 treatment (LPS + Tα1). **a** Principal component analysis (PCA) diagram of Control, LPS, and LPS + Tα1 groups of DPCs. **b** The number of differentially expressed genes (DEG) including up-regulated genes and down-regulated genes in LPS vs Control, LPS + Tα1 vs Control, and LPS + Tα1 vs LPS. **c** Venn diagram of DEGs of LPS vs Control, LPS + Tα1 vs LPS, and LPS + Tα1 vs Control. **d** Heatmap of 220 differentially expressed ferroptosis-related genes (DE-FRGs), from the intersected genes between 7307 DEGs (both in LPS vs Control and LPS + Tα1 vs LPS) and 471 ferroptosis-related genes including driver, suppressor, marker, and unclassified regulator (FerrDb V2 data). **e** 127 up-regulated and 93 down-regulated DE-FRGs were listed, and these genes reversely expressed after Tα1 addition

RNA sequencing (RNA-seq) was carried out to explore the mechanism by which thymosin α1 alleviates ferroptosis in LPS-stimulated DPCs. Principal component analysis (PCA) showed that PC1 (47.67%) accounted for the most variance in the inter-group disparity of DPCs treated with Tα1 and LPS. The dispersion of LPS-stimulated DPCs treated with Tα1 (LPS + Tα1) was similar to that of the control and differed from that of LPS-stimulated DPCs (Fig. 6a). Based on |log2(FoldChange)| ≥ 1 & adjusted p-value (padj) ≤ 0.05, 9 095 differentially expressed genes (DEGs) were identified in the comparison between LPS and Control, including 4 586 up-regulated genes and 4 509 down-regulated genes. Additionally, 8 584 DEGs were found in the comparison between LPS + Tα1 and LPS, comprising 4 319 up-regulated genes and 4 265 down-regulated genes. The number of DEGs between LPS + Tα1 and Control was significantly less (Fig. 6b). In Kyoto Encyclopedia of Genes and Genomes (KEGG) analysis based on padj ≤ 0.05, the top 20 significantly enriched signaling pathways were listed in LPS versus Control and LPS + Tα1 versus LPS (Figure. S3a, b). However, no significant enrichment of pathways was presented in LPS + Tα1 versus Control, and only the top 20 pathways with padj > 0.05 were shown (Supplementary Fig. 3c). In the Venn diagram, 7 307 DEGs were found in both LPS versus Control and LPS + Tα1 versus LPS. The DEGs were intersected with 471 ferroptosis-related genes including drivers, suppressors, markers, and unclassified regulators (FerrDb V2 data) (Fig. 6c). There were 127 up-regulated differentially expressed ferroptosis-related genes (DE-FRGs) and 93 down-regulated DE-FRGs displayed in LPS versus Control by a heatmap. The expression of DE-FRGs was reversed when Tα1 was added to LPS-stimulated DPCs (Fig. 6d, e). RNA-seq indicated that Tα1 may significantly reverse the ferroptosis of LPS-stimulated DPCs.

## Discussion

Exploring cellular heterogeneity and regulatory changes in inflammatory diseases is crucial for the development of medical therapies. Single-cell RNA sequencing has emerged as a powerful tool for studying inflammatory diseases.^31,32^ It enables a detailed analysis of individual cells, providing insights into the heterogeneity of cell types and states within a disease tissue. ScRNA-seq can reveal the heterogeneity of different cell types between healthy pulp and pulpitis. The number of fibroblasts showed a significant decrease in cell proportion in pulpitis, and the number of DEGs of fibroblasts in the two groups of pulp tissue was the largest. This suggests that the change in fibroblasts plays a crucial role in pulpitis. These findings help us understand the pathological changes of pulpitis and may lead to the development of targeted therapies.

Ferroptosis, an iron-dependent form of regulated cell death, has recently attracted significant attention in the field of biomedical research. A mounting body of evidence indicates that ferroptosis is implicated in the inflammatory response. Targeting ferroptosis holds great promise in the prevention and treatment of inflammatory diseases.^33^ FerrDb is the first global database that summarizes ferroptosis regulators and is often used to study the changes in genes related to ferroptosis and diseases. Our studies indicated that ferroptosis occurs in pulpitis as evidenced by DE-FRGs in each cell type, detecting low expression of GPX4 and high expression of PTGS2 in immunohistochemical assays, and increased ROS and ferrous level. Furthermore, in LPS-stimulated DPCs, ferroptosis occurrence is also shown by high level of ferrous, low expression of GPX4 and FTL proteins, and GSEA in RNA-sequencing of LPS versus Control.

GPX4 and PTGS2 play crucial and distinct roles in the mechanism of ferroptosis.^15,34^ GPX4, a key enzyme in the cellular antioxidant defense system, is a critical negative regulator of ferroptosis, protecting cells from oxidative damage by reducing lipid peroxides.^34,35^ It specifically reduces lipid hydroperoxides to their corresponding alcohols, thereby preventing the accumulation of toxic lipid peroxides. By eliminating lipid peroxides, GPX4 blocks the propagation of oxidative stress and subsequent cell membrane damage, which are hallmarks of ferroptotic cell death. PTGS2, also known as cyclooxygenase-2 (COX-2), act as a pro-ferroptotic factor under certain conditions, promoting lipid peroxidation and cell death through the production of prostaglandins and modulation of inflammatory and redox-related pathways. It can metabolize arachidonic acid and other polyunsaturated fatty acids into prostaglandin H2 (PGH2) and other prostaglandins. These metabolites can enhance lipid peroxidation by serving as substrates for further oxidative reactions or by modulating the activity of other enzymes involved in lipid metabolism.^36^ PTGS2 is often upregulated in response to inflammatory stimuli. In the context of inflammation, the increased activity of PTGS2 can lead to the production of pro-inflammatory prostaglandins, which can trigger a series of events that ultimately promote ferroptosis.^37,38^ Though PTGS2 did not show the different expression in ScRNA-seq of pulpitis vs healthy pulp, immunochemistry assay showed that PTGS2 high expressed in pulpitis. Single-cell sequencing is a powerful technique in biological research, but it has several limitations. Due to technical biases, low sequencing depth, spatial information loss, post-transcriptional regulation, cell heterogeneity, etc., gene expression prediction results of ScRNA-seq might be inconsistency with histological protein expression results.^39–41^ Therefore, it is important to consider these factors when interpreting the results of single-cell sequencing and to validate the findings using other experimental techniques.

To further explore the possible mechanism of ferroptosis in pulpitis, six KEGG signal pathways were sorted out. The high enrichment of oxidative phosphorylation was considered to have the closest relation with ferroptosis in pulpitis by scRNA-seq. Oxidative phosphorylation is a crucial process in cellular energy metabolism and has a significant impact on the occurrence of ferroptosis. Oxidative phosphorylation occurs in the mitochondria, which is a major source of ROS. Excessive activation of oxidative phosphorylation will cause a large amount of ROS production, lipid peroxidation, and finally trigger ferroptosis.^42,43^ Furthermore, oxidative phosphorylation activation will disrupt mitochondrial function, and affect iron homeostasis. Iron accumulation within the cell can enhance the fenton reaction, generating more hydroxyl radicals and promoting lipid peroxidation, thereby facilitating ferroptosis.^44^ Therefore, oxidative phosphorylation may contribute to ferroptosis occurrence in pulpitis.

Based on ferroptosis contribution to inflammation, we hypothesize that inhibiting ferroptosis in pulp inflammation may be a potential therapeutic strategy for pulpitis. By blocking the key steps of ferroptosis, such as iron accumulation, we may be able to reduce the production of ROS and inflammatory mediators, thereby alleviating the inflammatory response of pulpitis. In this study, a biologically active peptide, thymosin α1, is investigated to alleviate pulpitis by inhibiting ferroptosis. First, we evaluated the effect of Tα1 on inflammatory DPCs. Tα1 can decrease ferrous level and the expression of PTGS2 protein, and increase the expression of GPX4 and FTL proteins. Second, in RNA-sequencing, the addition of Tα1 reversed the expression of the majority of DE-FRGs of DPCs with LPS stimulation. Third, in the pulpitis model of rats, PTMA increased the expression of GPX4, decreased the expression of PTGS2, and reduced the infiltration of inflammatory cells. Additionally, Tα1 may significantly reduce the expression of inflammatory factors such as TNF-α, IL-1β, and IL-6. These findings indicated that Tα1 alleviated pulpitis possibly by inhibiting ferroptosis. Thymosin α1, a regulator of immune homeostasis, can play a role in controlling inflammation, immunity, and tolerance in various clinical settings.^45^ Thymosin α1 is commonly employed as an immune enhancer in various infectious diseases, including hepatitis B, hepatitis C, sepsis, severe acute pancreatitis, and acquired immune deficiency syndrome.^20,46,47^ Tα1 can regulate immune cells like T cells, B cells, macrophages, and natural killer cells through various Toll-like receptors.^20^ In this study, thymosin α1 was first investigated in inflammatory DPCs and evaluated for its application in pulpitis.

RNA-seq was used to explore the mechanism by which thymosin α1 influences ferroptosis in DPCs. In the comparison between LPS and Control, cellular senescence and p53 signaling pathways, which are related to ferroptosis, were enriched in the top 20 of KEGG.^48,49^ In the comparison between LPS + Tα1 and LPS, cellular senescence and p53 signaling pathways were also highly enriched in the top 20. It is possible that Tα1 affects the expression of these two signaling pathways to regulate ferroptosis, although oxidative phosphorylation is the most enriched pathway in pulpitis in scRNA-seq. Bulk RNA-seq differs from scRNA-seq that occurs in a complex dental pulp microenvironment. Bulk RNA-seq of DPCs analyzes a population of cells as a whole and provides an average view of gene expression patterns across a group of cells. Bulk RNA-seq of DPCs is only stimulated by LPS, unlike cells in pulpitis induced by multiple irritants. Thus, it is not surprising that some different KEGG pathways are generated in bulk RNA-seq of DPCs and scRNA-seq of dental pulp. Despite the differences, we detected data related to ferroptosis in both groups of RNA-seqs. Therefore, thymosin α1 inhibiting ferroptosis would alleviate inflammatory DPCs and pulpitis. Furthermore, though PTGS2 proteins showed the different expression between LPS + Tα1 and LPS groups, PTGS2 was not found the significant difference in RNA-seq of inflammatory DPCs with Tα1 treatment, and even FTL showed the contrary results between proteins expression and RNA-seq (Fig. 3b and Fig. 6e). The inconsistent results may be related with several reasons: mRNA stability, transcription factor activity, alternatively splicing, ribosome loading and translation efficiency, post-translational regulation, cell-cycle dependence, and environmental factors, etc.^50–53^

## Conclusions

Our study provides new insights into the mechanism of ferroptosis in pulp inflammation and the potential of thymosin α1 for the treatment of pulpitis. Although our findings indicated that thymosin α1 inhibits inflammatory dental pulp cells and pulpitis, further research is needed to clarify the effect of thymosin α1 on immune cells. This is because the number of T cells, B cells, macrophages, and plasma cells is significantly higher in pulpitis than in healthy pulp. Therapeutic strategies of thymosin α1 for pulpitis based on ferroptosis still need to be further studied.

## Materials and Methods

### Ethics and dental pulp sample collection

This study adhered to all relevant ethical regulations and was approved by the Medical Ethics Committee of the Hospital of Stomatology, Sun Yat-Sen University (Seal) (KQEC-2023–43-01). In this study, discarded teeth of patients were used with their informed consents. This study conformed to all relevant ethical requirements. We collected extracted third molars for single-cell RNA sequencing (scRNA-seq), immunohistochemical staining and dental pulp cells (DPCs) isolating and culture. At no point did our study have any influence on the fate of the teeth. Three healthy third molars without caries lesions from patients aged 18, 22, and 26 years and three pulpitis third molars from patients aged 28, 32, and 33 years were extracted. The samples were then transported to ice-cold phosphate-buffered saline (PBS, Hyclone, Logan, UT). Dental pulp tissues were isolated by splitting the teeth, and single-cell suspensions were prepared according to a previous study.^54^ The raw data of scRNA-seq of the six dental pulp samples were upload to the website address: https://www.ncbi.nlm.nih.gov/geo (Healthy control dental pulp: GSE274562 and Pulpitis: GSE280528).

### ScRNA-seq and secondary analysis of gene expression

The cell suspension is loaded into Chromium microfluidic chips and barcoded with a 10× Chromium Controller. RNA from barcoded cells is reverse-transcribed and sequencing libraries are constructed using a Chromium Single Cell 3’ v2 reagent kit. Sequencing is performed with Illumina. Raw reads are demultiplexed and mapped to the reference genome by 10× Genomics Cell Ranger pipeline. Downstream single-cell analyses are done using Cell Ranger and Seurat.^55,56^ For secondary analysis of gene expression, the Seurat package is used for normalization, dimensionality reduction, clustering, and differential expression analysis. CCA alignment method is employed for integrated analysis.^57^ Highly variable genes are selected for clustering. Differential expression analysis between samples is done using the edgeR package.^58^ The clusterProfiler R package is used to test the statistical enrichment of marker genes in KEGG pathways.^59^ Differentially-expressed genes (DEGs) analysis between healthy control dental pulp and pulpitis is conducted using the non-parametric two-sided Wilcoxon rank-sum test. Genes with a *P*-adjust value < 0.1 and |LogFC | > 10% are considered aged DEGs.

### Reactive oxygen species, ferrous ion and immunohistochemical staining of pulp tissue

Third molars without caries lesions from patients aged 21, 23, and 28 years and those with pulpitis from patients aged 30, 35, and 27 years were extracted, and the gingival tissue on the surface of the teeth was removed. The teeth were soaked in a 10% neutral buffered formalin solution for 1 d. The samples were decalcified in sodium ethylene diamine tetracetate acid (EDTA) solution for 4 wk. The EDTA was replaced every three days. The progress of decalcification was monitored by X-ray each week to avoid over-decalcification. After decalcification, the tooth samples were embedded using OCT and stored at −20 °C for 24 h. The samples were cut into 4 μm-thickness slices and baked at 37 °C for 1 h.

In the evaluation of reactive oxygen species (ROS) and ferrous ions, the slices were respectively added with the fluorescent probe dichlorodihydrofluorescein diacetate (DCFH-DA, Beyotime, China) or FerroOrange (DOJINDO, Japan), and incubated at 37 °C for 30 min. After the unbound DCFH-DA or FerroOrange were removed by washing and with absorbent paper, the sections were blocked using DAPI. Subsequently, the stained sections were observed and images were captured under a Zeiss LSM 980 laser scanning confocal microscope.

For immunohistochemical staining, the slices were first permeabilized with 0.3% Triton X-100 for 10 min and then with 3% H₂O₂ solution at room temperature for 20 min. Next, they were blocked with serum-based blocking buffer for 1 h. The slices were incubated overnight with primary antibodies against GPX4 (1:200, Cat# DF6701, Affinity Biosciences) or PTGS2 (1:200, sc-514489, Santa Cruz Biotechnology). After washing to remove unbound primary antibody, they were incubated with a secondary horseradish peroxidase-conjugated goat anti-rabbit antibody (ZSGB-Bio, China) for GPX4 or goat anti-mouse antibody (ZSGB-Bio, China) for PTGS2 at 37 °C for 1 h. After diaminobenzidine staining and hematoxylin counterstaining, the slices were dehydrated, cleared, and mounted in an Aperio AT2 image-capturing device. Imagescope software version 12.4.3 is used to capture scanned images.

### Dental pulp cells isolated, cultured and proliferation assay

Dental pulp tissue was collected from an extracted third molar of patient aged 23 years by splitting the tooth crown and then digested with collagenase type I (SG60001, BioFroxx) and dispase (Cat: D6430, solarbio). After digestion, the cell suspension was filtered to remove undigested tissue debris and obtain DPCs suspension. The filtered cell suspension was centrifuged, and the cell pellet was then resuspended in Alpha-mem complete medium (α-MEM, Gibco) supplemented with 10% fetal bovine serum (FBS, fetal bovine serum, Promocell) and antibiotics. The cells were cultured in a humidified incubator at 37 °C with 5% CO₂, and observed under a microscope to monitor their growth and morphology.

DPCs at passage 5 were seeded in 96-well plates at a density of 5 × 10³ cellsperwell, and placed in an incubator with 5% CO₂ at 37 °C for 24 h. After the medium was removed, DPCs were added with α-MEM containing 10% FBS and 0 μg/mL, 1 μg/mL, 2 μg/mL, or 4 μg/mL of thymosin α1 (Tα1, ZADAXIN, SciClone Pharmaceuticals, Inc), and further incubated in an incubator with 5% CO₂ at 37 °C for 12 h, 24 h, and 48 h, respectively. The medium was removed, and 100 μL of serum-free medium and 10 μL of CCK8 (Cell Counting Kit-8, Tongren Chemical) were added to each well. The DPCs were cultured in an incubator with 5% CO₂ at 37 °C in the dark for 2 h. The optical density (OD) value of each well was detected and read at an absorbance of 450 nm.

### Construction of *Ptma* gene silenced dental pulp cells by lentiviral transfection

DPCs at passage 2 were seeded in T25 bottles at a density of 5 × 10⁵ cells per well. The cells grew to a 30%-50% confluency in α-MEM supplemented with FBS. The medium was replaced with 5 mL of fresh α-MEM containing 5 μg/mL polybrene. Then, 100 μL of the lentivirus particles carrying 1 × 10⁸ transduction units (Tu)permL shRNA targeting the *Ptma* gene was added to the cell culture medium. The transfected cells were incubated overnight. After transfection, the medium was replaced with fresh medium containing 2 μg/mL puromycin and then incubated for 7 d until untransfected cells were eliminated. Control shRNA without carrying any gene was also transfected into DPCs. The reduction in PTMA mRNA and protein expression in DPCs verified the success of *Ptma* gene silencing by reverse transcription quantitative polymerase chain reaction (RT-qPCR) and western blot.

### Western blot

Western blot was employed to assess the expression of ferroptosis-related proteins in DPCs. DPCs at passage 5 and shPTMA-DPCs were seeded in 6-well plates at a density of 1 × 10^6^ cells per well and incubated at 37 °C for 24 h. DPCs were designed into four groups. Control group: DPCs without any stimulation; LPS group: DPCs were stimulated with 10 μg/mL lipopolysaccharide (LPS from *porphyromonas gingivalis*, Invivogen, France); LPS + Tα1 group: DPCs were treated with 10 μg/mL LPS and 1 μg/mL thymosin α1; LPS + shPTMA: shPTMA-DPCs were stimulated with 10 μg/mL LPS. The four groups of DPCs were cultured at 37 °C for 24 h, and total proteins were extracted using a bicinchoninic acid protein assay kit (Beyotime, Shanghai, China) in accordance with the manufacturer’s instructions. Proteins were separated and electrotransferred to PVDF membranes. After being blocked, the membranes were incubated with primary antibodies overnight at 4 °C, including GPX4 (1:1 000, #DF6701, Affinity, USA), FTL (1:5 000, Cat# 10727-1-AP, Proteintech), PTGS2 (1:1 000, AF7003, Affinity, USA), and ß-actin (1:1 000, #4970, Cell Signaling Technology, Danvers, MA, USA), and were then incubated with secondary antibodies. Protein bands were captured under an ImageQuant LAS 4000 mini imaging system.

### Quantitative real-time polymerase chain reaction

To investigate the expression of inflammatory factors in DPCs, total RNA in the four groups of DPCs: Control, LPS, LPS + Tα1, and LPS + shPTMA, were extracted via a RNA extraction kit (ES Science, China), and cDNA was generated by reverse transcription from the extracted RNA using PrimeScript Reverse Transcriptase (TaKaRa, Japan). *TNF-α*, *IL-1β*, and *IL-6* genes were evaluated by real-time PCR using Hieff qPCR SYBR Green Master Mix (LOW ROX, Yeasen, China) in a Roche LightCycler96. Human β-actin was considered a housekeeping gene. The primers are listed as follows: *TNF-α*: F: CCTCTCTCTAATCAGCCCTCTG, R: GAGGACCTGGGAGTAGATGAG; *IL-1β*: F: ATGATGGCTTATTACAGTGGCAA, R: GTCGGAGATTCGTAGCTGGA; *IL-6*: F: ACTCACCTCTTCAGAACGAATTG, R: CCATCTTTGGAAGGTTCAGGTTG; *β-actin*: F: TGACGTGGACATCCGCAAAG, R: CTGGAAGGTGGACAGCGAGG.

### Assay of ferrous ion and JC-10 of DPCs

To evaluate the effect of Tα1 on ferroptosis of inflammatory DPCs, we performed assay of ferrous ion and JC-10 of DPCs. DPCs were divided into four groups. Control group: DPCs without any stimulation; LPS group: DPCs were stimulated with 10 μg/mL LPS for 24 h; LPS + Fer-1 group: DPCs were first stimulated with 10 μg/mL LPS for 24 h, and then added 5 μmol/L Ferrostatin-1 (Fer-1, HY-100579, MCE) for another 24 h incubation; LPS + Tα1 group: DPCs were first stimulated with 10 μg/mL LPS for 24 h, and then added 1 μg/mL Tα1 for another 24 h incubation. The four groups of DPCs were treated with 1 μmol/L FerroOrange (F374, DojinDo, Kumamoto, Japan) and 10 μg/mL Hoechst 33342 at 37 °C for 30 min, according to the manufacturer’s protocol. The images were captured under a Zeiss LSM 980 and analyzed using ImageJ 1.43b software. Furthermore, the four groups of DPCs were collected by trypsin digestion and centrifugation. Subsequently, the DPCs were stained in the JC-10 staining buffer (40707ES03, Yeasen, Shanghai, China) at 37 °C for 20 min according to the manufacturer’s protocol. Flow cytometry was performed on the DPCs, and quantification was done using Image J Pro software. Spectrofluorometric analysis was carried out by detecting red-fluorescence JC-10 aggregates with 540-nm excitation and 590-nm emission and green fluorescence with 490-nm excitation and 525-nm emission. The ratio of red/green fluorescence intensity is used to evaluate the changes in mitochondrial membrane potential.

### In vivo pulpitis model of rats

Rat pulpitis models were employed to test whether PTMA alleviated pulpitis. The procedures of all animal experiments conformed to the Animal Ethics Procedures and Guidelines of the People’s Republic of China, and were approved by the Institutional Animal Care and Use Committee of Sun Yat-Sen University (Approval No: SYSU-IACUC-2024-002797). Male Sprague Dawley rats (6 weeks old, approximately 220 g/rat) were anesthetized by intraperitoneal injection of 1% sodium pentobarbital (0.1 mL per 10 g). The right mandibular first molars pulp were mechanically exposed using a high-speed 1/4 round bur on a portable dental unit. Rat pulpitis models were divided into three groups (3 rats per group). LPS group: a sterile gelatin sponge with an adsorption of 5 μL (10 μg/mL) LPS were placed on the exposed pulp; LPS-P(gs) group: a sterile gelatin sponge with an adsorption of 500 ng PTMA was placed above the LPS gelatin sponge; LPS-P(i) group: LPS gelatin sponge were placed on the exposed pulp, and 5 μL (100 μg/mL) PTMA were then injected into pulp cavity using a microliter syringe. The cavity was sealed with a flowable resin (ShoFu, Kyoto, Japan). The flowable resin in the access cavity was decreased to avoid the loss of resin. The left intact mandibular first molars pulp were considered a control group. All animals were treated with respect and in a species-appropriate manner. Their requirements for transport, accommodation, environmental conditions, nutrition, and veterinary care were fulfilled. No variations in weight gain or behavioral changes were observed. The animals were housed in an environment with free access to water and feed, under a 12-hour light-dark cycle at a temperature ranging from 20 to 25 °C.

### Assay of ferrous ion and reactive oxygen species in rat pulpitis

After 3 d, the rats were sacrificed. The mandibular bones were dissected, separated, and soaked in 4% paraformaldehyde. Mandibular bone samples in which there was loss of flowable resin in the access cavities were excluded. Mandibular first molars samples of control, LPS, LPS-P(gs), and LPS -P(i) groups were dissected, decalcified, embedded, sectioned, baked, and then evaluated Fe^2+^ and ROS level as described in above assay of Fe^2+^ and ROS of human pulpitis sample.

### Haematoxylin and eosin and immunohistochemical staining of GPX4 and PTGS2 in rat pulpitis

Four groups of rat tooth pulp slices were incubated with primary antibodies against GPX4 or PTGS2, secondary antibody, and stained with DAPI, as described in the above preparation of human pulpitis sample slices for immunohistochemistry of GPX4 and PTGS2. Furthermore, the slices were subjected to haematoxylin and eosin (H&E) staining. Images were captured using an Aperio AT2 image-capturing device. GPX4 and PTGS2 positive cells were quantified using Image J Pro software.

### Total RNA sequencing of LPS-stimulated DPCs with Tα1 treatment

DPCs were divided into control group, LPS group, and LPS + Tα1 group, as described in the above western blot assay. Total RNA were isolated in three groups, and mRNA is purified from the total RNA via poly-Toligo-attached magnetic beads and random fragmentation was carried out. RNA integrity was evaluated using the RNA Nano 6000 Assay Kit of the Bioanalyzer 2100 system (Agilent Technologies, CA, USA). Each group were repeated five times. Library construction and sequencing, RNA-seq data analysis process, identification of DEGs, and enrichment of KEGG were performed based on the procedures Novogene Bioinformatics Technology Co., Ltd (Beijing, China) provided. DEGs of LPS vs Control were intersected with ferroptosis gene regulators including driver, suppressor, marker, and unclassified regulator in FerrDb database (http://www.zhounan.org/ferrdb/current/).

### Statistical analysis

All statistical analyses of scRNA-seq data were performed using R 3.5.2 (https://www.R-project.org). The in vitro and in vivo experiments were repeated independently, and similar results were obtained at least three times. Statistical analyses were conducted by GraphPad Prism software (v9.2). Statistical significance was set at *P* < 0.05.

## Supplementary information

Supplementary figures

Ethical Approval

## Data Availability

The data that support the findings of this study are available from the corresponding author upon reasonable request.
