# Supplementary material for: Thymosin α1 alleviates pulpitis by inhibiting ferroptosis of dental pulp cells
Source: Int J Oral Sci. 2025 Oct 14;17:68. doi: 10.1038/s41368-025-00394-4 (PMC12521540; doi:10.1038/s41368-025-00394-4)
Supplement: Supplementary file 1 — Supplementary figures [file 41368_2025_394_MOESM1_ESM.docx]

**Supplementary figures**


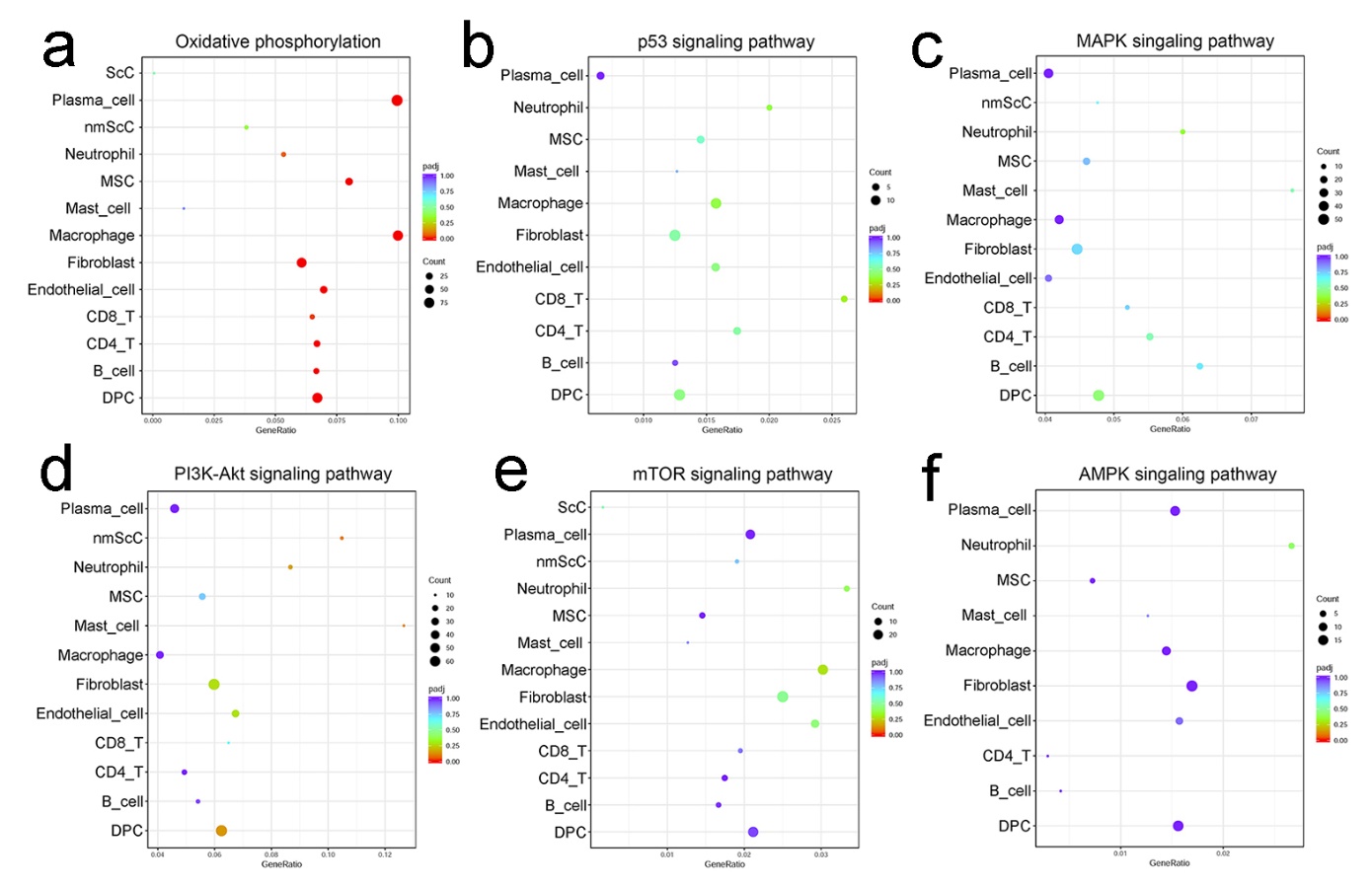
Fig. S1. KEGG enrichment analysis of ferroptosis in single-cell RNA sequencing between pulpitis and healthy pulp tissue. (a) oxidative phosphorylation; (b) p53 signaling pathway; (c) MAPK signaling pathway; (d) PI3K-Akt signaling pathway; (e) mTOR signaling pathway; (f) AMPK signaling pathway.


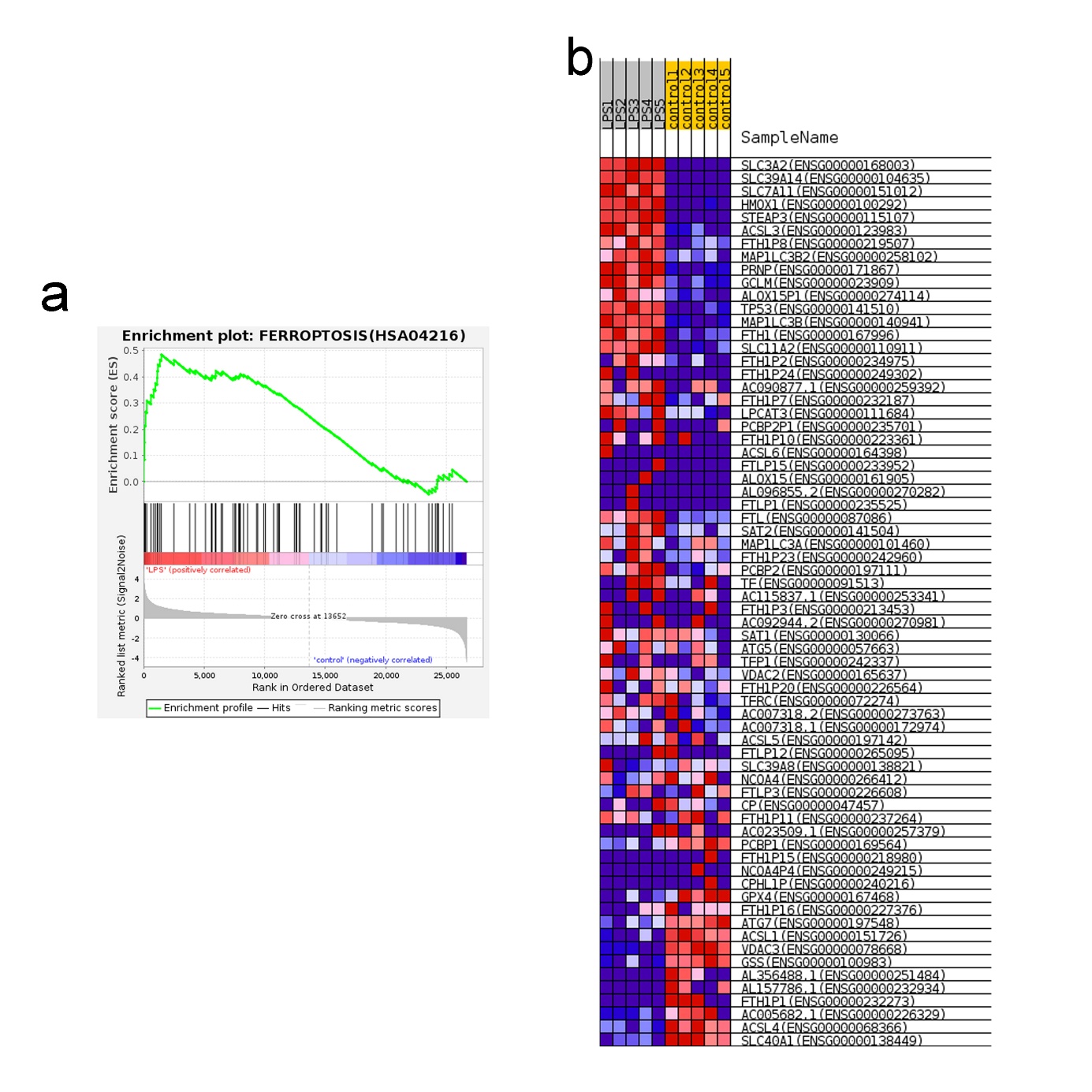
 Fig. S2. (a) Gene set enrichment analysis (GSEA) of ferroptosis in LPS-stimulated DPCs versus control DPCs. (b) Heatmap of different gene expression in GSEA of ferroptosis in LPS-stimulated DPCs versus control DPCs.


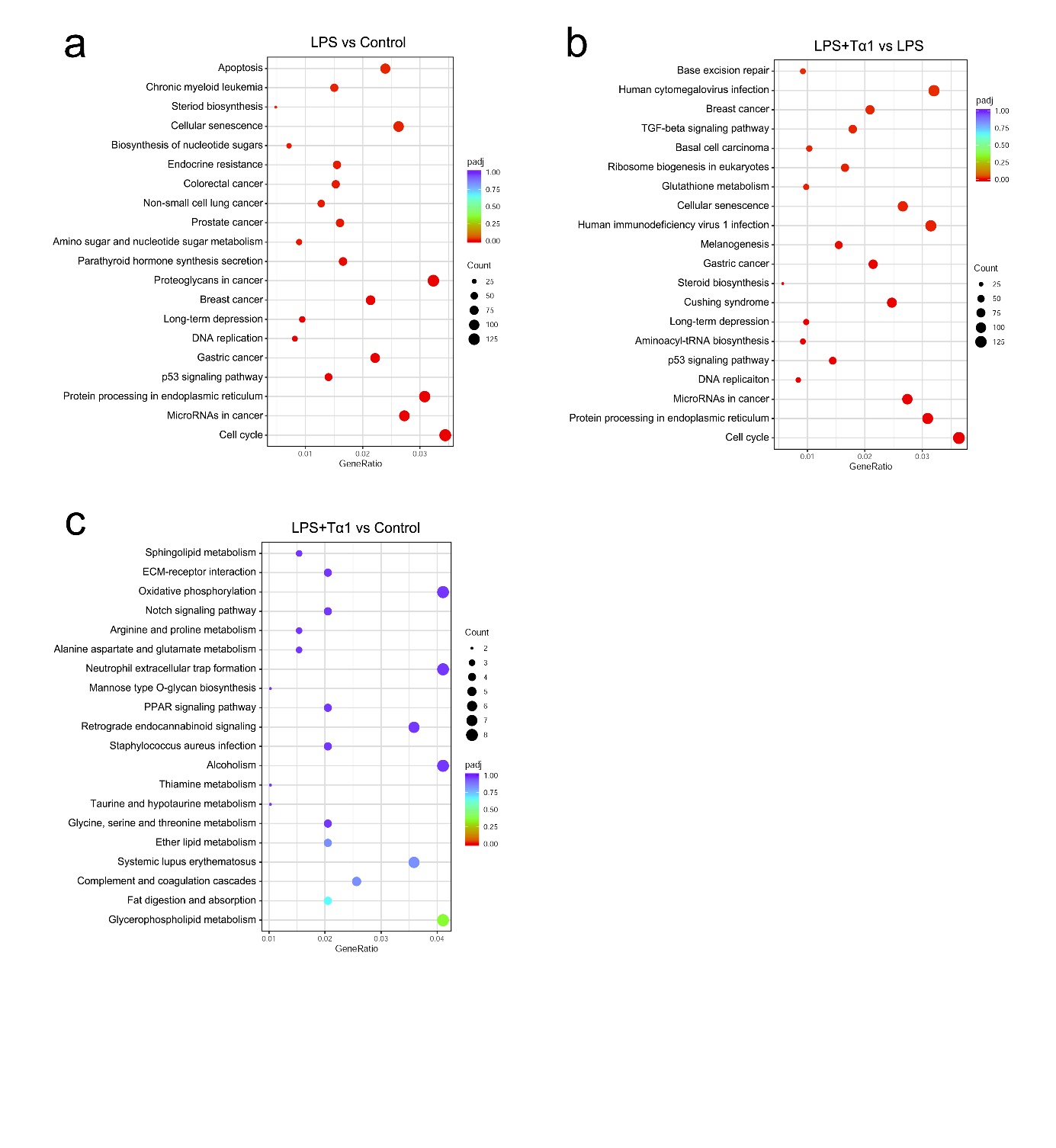
 Fig. S3. RNA-seq of dental pulp cells (DPCs) with LPS stimulation and thymosin α1 treatment. RNA-seq of DPCs were divided into three groups: Control, LPS-stimulated DPCs (LPS), and LPS-stimulated DPCs with thymosin α1 treatment (LPS+Tα1). Top 20 KEGG enrichment pathway in LPS vs Control (a), LPS+Tα1 vs LPS (b), and LPS+Tα1 vs Control (c).
