# Supplementary material for: Thymosin α1 alleviates pulpitis by inhibiting ferroptosis of dental pulp cells
Source: Int J Oral Sci. 2025 Oct 14;17:68. doi: 10.1038/s41368-025-00394-4 (PMC12521540; doi:10.1038/s41368-025-00394-4)
Supplement: Supplementary file 2 — Ethical Approval [file 41368_2025_394_MOESM2_ESM.pdf]

中山大学实验动物管理与使用委员会
Institutional Animal Care and Use Committee,
Sun Yat-Sen University

中山大学动物实验伦理审查同意书

Affidavit of Approval of Animal Use Protocol, IACUC, SYSU

申请编号 Application No.	2024002461	批准编号 Approval No.	SYSU-IACUC-2024-002797

本动物实验方案经过中山大学实验动物伦理委员会审核，符合动物保护、动物福利和伦理原则，符合国家实验动物福利伦理的相关规定。The animal use protocol listed below has been reviewed and approved by the Institutional Animal Care and Use Committee (IACUC), Sun Yat-Sen University.

实验名称 Protocol Title	大鼠牙髓炎模型建立<br>Establishment of rat pulpitis model				
实验申请人 Applicant	吴洁<br>Wu jie	职称/学位 Title/Degree	硕士<br>Master	邮箱 Email	2254639236@qq.com
实验负责人 Principle Investigator (PI)	郑金绚<br>Zheng Jinxuan	职称/学位 Title/Degree	博士后<br>Post-Doctor	邮箱 Email	418702395@qq.com
院系(部门) Department	中山大学光华口腔医学院<br>Guanghua School of stomatology, Hospital of stomatology, Sun Yat-sen University	申请日期 Application date	2024/10/6		
动物种系 Species or Strains	大鼠 CD(SD)IGS<br>CD(SD)IGS	动物数量 Quantity	60		
计划执行时间 Period of Protocol	2024/6/25 ~2024/9/25	实验动物使用许可证 Number of Animal use permit	中山大学（实验动物中心何母楼北 EFGH 区）（SYXK（粤）2024-0081）		
审查意见 Results of inspection	☐符合动物福利伦理要求，同意实验 **Agree**<br>☑调整方案后，可进行实验 **Agree after modification**				
兽医师 Chief Veterinary Officer	[signature]	日期 Date	2024-11-01		

中山大学实验动物管理与使用委员会 IACUC SYSU

中山大学实验动物伦理委员会（IACUC，SYSU）

主席（Chairman）： [signature]

日期（Date）： 2024-11-01

地址: 广州中山二路 74 号中山大学实验动物中心　邮编：510080
Add: Laboratory Animal Center, SUN YAT-SEN UNIVERSITY, No. 74，Zhongshan Road II, Guangzhou, 510080, P.R. China
